# Human Stool Preservation Impacts Taxonomic Profiles in 16S Metagenomics Studies

**DOI:** 10.3389/fcimb.2022.722886

**Published:** 2022-02-08

**Authors:** Anne Plauzolles, Eya Toumi, Marion Bonnet, Guillaume Pénaranda, Ghislain Bidaut, Laurent Chiche, Jérôme Allardet-Servent, Frédérique Retornaz, Benoit Goutorbe, Philippe Halfon

**Affiliations:** ^1^ Clinical Research and R&D Department, Laboratoire Européen Alphabio, Marseille, France; ^2^ MEPHI, IHU Méditerranée Infection, Aix Marseille Université, Marseille, France; ^3^ CRCM, Aix‐Marseille Univ U105, Inserm U1068, CNRS UMR7258, Institut Paoli‐Calmettes, Marseille, France; ^4^ Infectious and Internal Medicine Department, Hôpital Européen Marseille, Marseille, France; ^5^ Intensive Care Unit, Hôpital Européen Marseille, Marseille, France; ^6^ Université Paris-Saclay, INRAE, MaIAGE, Jouy-en-Josas, France

**Keywords:** microbiota, standardization, 16S metagenomics, human gut, preservation, stool, stabilizing solution.

## Abstract

Microbiotas play critical roles in human health, yet in most cases scientists lack standardized and reproducible methods from collection and preservation of samples, as well as the choice of omic analysis, up to the data processing. To date, stool sample preservation remains a source of technological bias in metagenomic sequencing, despite newly developed storage solutions. Here, we conducted a comparative study of 10 storage methods for human stool over a 14-day period of storage at fluctuating temperatures. We first compared the performance of each stabilizer with observed bacterial composition variation within the same specimen. Then, we identified the nature of the observed variations to determine which bacterial populations were more impacted by the stabilizer. We found that DNA stabilizers display various stabilizing efficacies and affect the recovered bacterial profiles thus highlighting that some solutions are more performant in preserving the true gut microbial community. Furthermore, our results showed that the bias associated with the stabilizers can be linked to the phenotypical traits of the bacterial populations present in the studied samples. Although newly developed storage solutions have improved our capacity to stabilize stool microbial content over time, they are nevertheless not devoid of biases hence requiring the implantation of standard operating procedures. Acknowledging the biases and limitations of the implemented method is key to better interpret and support true associated microbiome patterns that will then lead us towards personalized medicine, in which the microbiota profile could constitute a reliable tool for clinical practice.

## Introduction

Over the past decade, an increasing number of studies have been published focusing on the human microbiome. While there is no doubt that these findings have helped us better comprehend the complexity of our microbiome and its implications on our health, the scientific community must compose with studies sometimes showing contradictory findings most often due to variabilities in protocols, cohorts’ characteristics and sizes. The lack of standards hampers our expertise, as studies show inconsistencies, often resulting from technological bias rather than a true biological signature. To utilize microbiome science to its full potential, technical and computational methods must be standardized, and quality controls must be implemented to transition in the near future from a basic research environment to the clinic.

It is now common knowledge that our microbiome colonizes all body surfaces, especially our gut microbiome, which strongly impacts nearly every aspect of host physiology (Sekirov et al., 2010; Lozupone et al., 2012; Sommer and Bäckhed, 2013). Multiple lines of evidence now link alterations in the gut microbiome to numerous diseases (Ley et al., 2006; Sartor, 2008; Wen et al., 2008; Sekirov et al., 2010; Cryan and Dinan, 2012; Louis et al., 2014). However, microbiome studies most often lead to mixed results, halting our progress and hindering potential diagnosis, disease prediction and therapeutic intervention of microbiome analyses. Hence, most individual bacteria are not consistently associated with a given disease. Such discrepancies, in regard to microbiome signature patterns, are likely due to heterogeneity across study populations (small size, genetic factors, lifestyle) or the studied model or could be influenced by methodological differences among studies (Nguyen et al., 2015; Costea et al., 2017; Hornung et al., 2019).

The reality of microbiome research is that a variety of biological and technical factors can impact the quality of samples and their microbial content (Kim et al., 2017). The gut microbiome is the most challenging human ecosystem to characterize due to its heterogeneous bacterial populations. Its composition varies widely from one individual to another and involves a majority of bacterial populations that are very sensitive to oxygen (Conrads and Abdelbary, 2019), as well as remnants of human and food DNA and inhibitors likely to hamper subsequent analytical steps (Nechvatal et al., 2008). Technical bias can then result in misleading findings and can affect the quality of the data. Throughout the series of steps that a fecal sample undergoes to identify and characterize its microbial content, sampling and stabilization are key in the pre-analytical protocol and can heavily impact data quality. Previous studies have demonstrated that storage conditions of stool samples have only a small impact on their microbial content (Roesch et al., 2009; Lauber et al., 2010); however, more recent findings show otherwise (Cardona et al., 2012; Choo et al., 2015; Gorzelak et al., 2015; Guo et al., 2016; Hickl et al., 2019). DNA and RNA deteriorate rapidly after collection when kept at room temperature (Cardona et al., 2012), while the chemistry of existing stabilizing solutions has also demonstrated an impact on the recovery of genomic microbial content, resulting in a source of bias (Wu et al., 2019; Chen et al., 2020). Despite these conflicting results and challenges, a few principles are currently well acknowledged by the scientific community: avoid freeze-thaw cycles and temperature fluctuations throughout the preservation process (Cardona et al., 2012; Gorzelak et al., 2015; Thomas et al., 2015; Kim et al., 2017)^;^ when possible, shorten the transportation time; and freezing samples at -20°C or -80°C provides an optimal solution when immediate analysis of fresh sample is not an option (Wu et al., 2010; Bahl et al., 2012; Carroll et al., 2012; Fouhy et al., 2015; Gorzelak et al., 2015; Hale et al., 2015; Voigt et al., 2015; Shaw et al., 2016; Sinha et al., 2016; Song et al., 2016; Hickl et al., 2019; Wu et al., 2019).

In regard to studying microbiome composition using metagenomics, the method of collection that yields the most accurate results involves analyzing samples immediately after collection. However, this can be logistically challenging for samples such as stools that cannot be produced on demand. Any stabilizing method induces rapid changes in the presence and/or abundance of certain bacterial populations (Song et al., 2016). Despite different efficacies in stabilizing the true biological profile, the preservation step can result in biases even during short-term storage, but these alterations are, for most commonly utilized solutions, smaller or comparable to differences among technical replicates. Technical variability, albeit smaller than interindividual variability, may obscure subtle and meaningful alterations. Therefore, the choice of stabilization is highly dependent on factors such as limitations, availability, ease of use, cost and compatibility with the study’s goals and/or ‘omics’ methods.

While the lack of standards affects the microbiome field in every ‘omic’ science and their related testing phases, including pre-analytical, analytical and post-analytical steps in sample processing, our research here focuses on technical bias in the pre-analytical handling of fecal samples in the study of gut microbiota through 16S metagenomics. For the past few years, the lack of standards and the sources of errors in datasets have been highlighted in the literature. Emerging protocols have arisen, but comparative studies, including comparison of most recent DNA stabilizers, are not sufficient to fully understand the bias that can emerge during this step. Our study aimed to evaluate and compare a large panel of stabilizing solutions that are either widely used by the scientific community or suited to the collection of fecal material. We also investigated the dynamic alterations that occurred over time in our samples based on their bacterial content and related phenotypical characteristics. Based on these results, acknowledging and identifying the limitations of DNA preservation could promote comparability among metagenomics studies and lead to clear guidelines that will be critical for scientific discovery going forward in understanding human microbiomes.

## Materials and Methods

### Stool Collection and Ethic Approval

Fecal samples were collected from 15 French volunteers (11 women and 4 men) between 20 and 46 years old. Most samples (n=10) were collected in the laboratory and handled immediately after defecation, while a minority (n=5) were collected at home and returned to our laboratory within 3 hours post-defecation. No medical records were collected. Age, gender and the sampling date were the only information provided by each volunteer.

All subjects provided written informed consent prior to participating in the study. These samples were anonymized and treated according to medical ethical guidelines.

### Stool Conservation Study Design

To provide a standardized protocol for fecal sampling and preservation, fecal samples were collected from 15 French volunteers (n=15).

**Figure 1:**
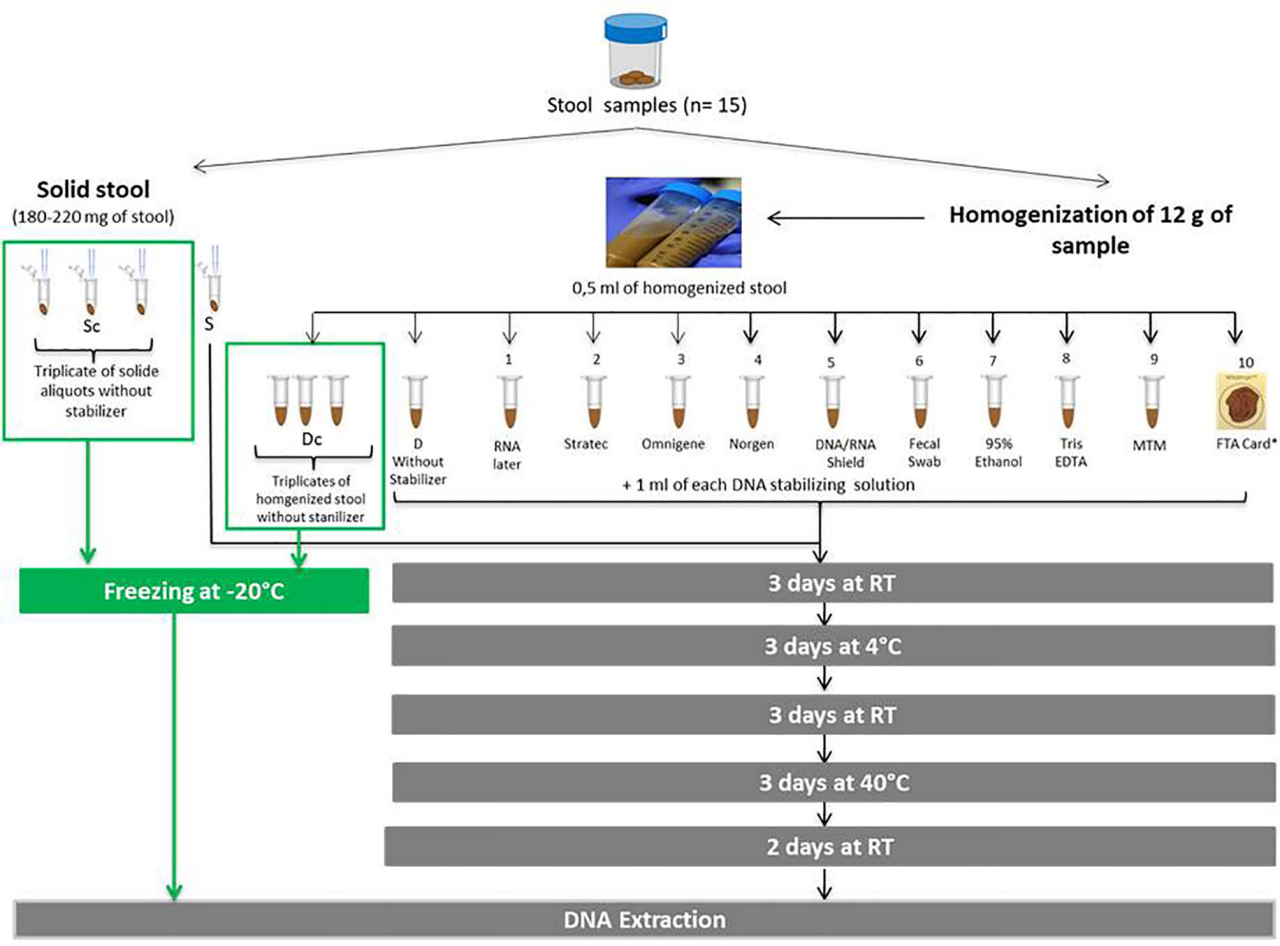
Illustration of the experimental protocol. Evaluation of ten commercial DNA stabilizing solutions for the storage of fecal samples. (Sc, frozen solid sample; Dc, frozen homogenized sample; S, solid sample without stabilizer; D:, homogenized sample without stabilizer; RT, room temperature). * The whatman FTA card is a cotton-based cellulose matrix containing chemicals that lyse cells, denature proteins and protect DNA; 0.5 ml of homogenized solution was dispatched directly onto the card.

A total of 12 aliquots were evaluated over a period of 14 days. Ten aliquots were mixed with a stabilizer while two (one solid, one homogenized) were mixed with ultrasterile water and served as controls representatives of an unstabilized sample. Beforehand, fecal homogenization of the collected stool was performed. to limit variability among aliquots, allowing for better evaluation of the impact of the stabilizing solutions tested. In parallel, to evaluate the interaliquot variability, two sets of triplicates were compared, one set of solid stool aliquots and another set of homogenized stool aliquots. The immediate freezing of the homogenized set (Dc) allowed to preserve the microbial profile of the fecal sample at time of collection as freezing prevents the proliferation and deterioration of the microbial entities that compose a fecal sample. Hence an average of the Dc’s triplicate was used as a reference profile in comparison to the aliquots preserved with a DNA stabilizer in order to evaluate the efficacy of the different stabilizers tested. Fecal homogenization was performed as follows: 12 g of stool was gently mixed with 30 ml of ultrasterile water for a few minutes. The collected stool was subsampled into 0.5 ml homogenized aliquots or 180-220 mg solid aliquots. All aliquots (solid and homogenized) were performed simultaneously and mixed with either a 1 ml of DNA stabilizer (for 10 aliquots, 1 to 10), or ultrasterile water (for two unstabilized controls, S and D) or immediately frozen at -20°C (for the two sets of triplicates, Sc and Dc). A total of 10 stabilizing solutions were tested: RNAlater (Ambion, Austin, US), Tris-EDTA (10 mM Tris-HCl pH 8.0, 1 mM EDTA) (Thermo Fisher Scientific, Massachusetts, US), 95% ethanol (VWR international, Pennsylvania, US), PrimeStore MTM (Longhorn Vaccines and Diagnostics, San Antonio, US), Stratec (Stratec Molecular GmbH, Berlin, Germany), OMNIgene-Gut (DNA Genotek, Ontario, Canada), Norgen (Norgen Biotek Corp., Thorold, Canada), DNA/RNA Shield (Zymo Research, Freiburg, Germany), Fecal Swab (Copan Italia S.P.A., Brescia, Italy), and Whatman FTA card (GE Healthcare Life Sciences, Illinois, US). For the FTA card method, a 0.5 ml sample was dispatched directly on the card. All stabilizers were tested on 15 fecal samples, except for PrimeStore MTM solution, which was tested on only 13 samples. All aliquots were preserved over a period of 14 days (**Figure 1**). Briefly, non-frozen aliquots (S, D and aliquots 1 to 10) were incubated for 14 days at varying temperatures fluctuating from 4°C to 40°C according to the following cycle: 3 days at room temperature (RT, approximately 25°C), 3 days at 4°C, 3 days at RT, 3 days at 40°C and 2 days at RT. These temperature fluctuations allowed evaluation of the efficacies of each stabilizing solution in harsh conditions. The temperature range chosen includes temperatures that a sample can be subjected to during transportation to the laboratory throughout the seasons for most countries worldwide.

Although, the collection of the fifteen samples was not uniform (either collected at home or in the laboratory), the reference for all sample was the profile at the time of stabilization. In this study, the alteration of all samples was compared to the reference samples (i.e., homogenized and immediately frozen samples stored with no additives, Dc).

### Stool DNA Extraction

Bacterial DNA was isolated from all stool aliquots using the NucleoSpin^®^ DNA Stool kit (Macherey-Nagel, Duren, Germany) following the manufacturer’s instructions. Extracted DNA was stored at -20°C until subsequent application.

### DNA Quantification and Purity Measurements

DNA quantification and purity (A260/A280 ratio) measurements were performed by spectrophotometry using a Nanodrop ND-1000 (Thermo Fisher Scientific, Massachusetts, US).

### 16S rRNA Gene Amplification, Library Preparation and High-Throughput Sequencing

To determine the bacterial composition of each aliquot, a 16S metagenomic sequencing library was created following Illumina’s recommendations (Illumina, 2013). Briefly, this protocol targets the V3-V4 regions of the 16S rRNA gene during a first PCR using specific primers with overhang adapters: 16S Amplicon PCR Forward Primer 5’ TCGTCGGCAGCGTCAGATGTGTATAAGAGACAGCCTACGGGNGGCWGCAG and 16S Amplicon PCR Reverse Primer 5’ GTCTCGTGGGCTCGGAGATGTGTATAAGAGACAGGACTACHVGGGTATCTAATCC. Resulting amplicons were then purified using Agencourt AMPure XP magnetic beads (Beckman coulter, Brea, US). Subsequently, a second PCR was performed from the purified PCR amplicons to attach dual indices and Illumina sequencing adapters using the Nextera XT Index Kit (Illumina, San Diego, US). Following a second purification with Agencourt AMPure XP magnetic beads, the PCR products were then checked with quality controls using a fragment analyzer (Agilent Technologies, California, US) and Qubit (Thermo Fisher Scientific, Massachusetts, US) to evaluate DNA fragment sizes and DNA concentrations of the purified products. Barcoded amplicons were pooled in equal concentrations to generate a 4 nM library. The pool of samples was denatured to a final concentration of 12 pM and combined with 5% PhiX control (Illumina, San Diego, US). The 16S rRNA gene libraries were sequenced using a MiSeq instrument (Illumina, San Diego, US).

### Experimental Validation

Demultiplexed and high-quality sequences (average quality score >Q30) were retrieved. Five samples with fewer than 30,000 reads were discarded, due to low quality DNA. All five samples were stabilized with Stratec Solution. A clustering analysis was performed to validate the experiment. Three samples were excluded from our analysis as they did not cluster with their technological replicates (**Supplementary Figure S6** for details).

### Bioinformatics Processing

Reads were processed using QIIME 2 (Bolyen et al., 2019) (version 2019.1.0) and its DADA2 (Callahan et al., 2016) plugin (q2dada2, version 2019.1.0). Preprocessing parameters were tuned to our dataset’s specifications: reads were trimmed at their 3’ ends at 245 bp, and reads shorter than this threshold were discarded; to remove amplification primers, 5’ trimming was performed at 17 bp and 21 bp for forward and reverse reads, respectively. Reads that exceeded the 2 sequencing errors expected were discarded, and chimera removal was performed with the consensus method of DADA2. A denoising step was performed, and amplicon sequence variants (ASVs) were collected in a counting table. Taxonomic assignment was performed using Kraken (Wood and Salzberg, 2014) (version 1.1) based on the NCBI RefSeq Targeted Loci database, which contains over 21,000 bacterial and archaeal 16S reference sequences covering more than 15,000 species. Kraken approach, initially designed for shotgun metagenomics, was proved to be efficient for 16S reads when adapting the reference database used (Lu and Salzberg, 2020). Even if direct support for 16S databases was made available with Kraken 2, both versions share the same core concepts, with an enhancement of memory and time efficiency in the latest (Lu and Salzberg, 2020).

### Statistical Analysis

Statistical analysis was performed with R (version 3.4) using the phyloseq package (McCurdie and Holmes, 2013) (version 1.22.3). Only ASVs with proportion beyond 10^–4^ in at least 5% of the samples were considered for the analysis. Beta diversity was assessed with several metrics: Jaccard and Bray-Curtis dissimilarity indices were computed based on rarefied data (35 395 reads per sample were used for rarefaction), while Aitchison’s distances (Gloor et al., 2017) were computed based on centered log-ratio transformed data with pseudo counts set at 0.5. Only the Bray-Curtis based analysis is shown, but different metrics confirmed this result (**Supplementary Figures S2**–**S4**).

First, the impact of homogenization was assessed by comparing mean distances within technological Sc (not homogenized) and Dc (homogenized) replicates across all stool samples collected using a paired Wilcoxon test.

Second, we evaluated stabilization performance by measuring the distance between each sample and its reference, defined as the barycenter of the ‘Dc’ replicates for the corresponding stool sample. We used a Kruskal-Wallis test to highlight the effect of the stabilizing solution on preservation of the bacterial content over storage time. Afterward, we performed a pairwise paired Wilcoxon test with Benjamini-Hochberg p-value correction for multiple hypothesis testing to determine which solutions performed better than others.

Finally, we searched for differentially abundant taxa, at the phylum and genus levels, between reference and stabilized samples with a Wilcoxon test, and p-values were adjusted with the Benjamini-Hochberg procedure. Furthermore, we gathered phenotypic data for the top 50 genera, representing up to 94% of all organisms found, regarding their oxygen sensitivity and their Gram stain status, as these characteristics are often conserved at the genus level (Schmaljohn and McClain, 1996; Brenner et al., 2005; Lowy, 2009). We used the LPSN database (Parte, 2018) to identify reference articles describing the characteristics of each genus. Gram stain status was defined as positive, negative or variable, and oxygen sensitivity was defined as strictly aerobic, strictly anaerobic, facultative anaerobic or microaerophile. Data are provided in **Supplementary Table T1**. We then clustered genera based on their median log2-fold change between stabilized samples and references using L2 distances and Ward’s linkage to identify genera that behaved similarly in the stabilizing solutions tested. To track potential links between genus phenotype and behavior in stabilizing solutions, we performed a χ² test for independence of categorical variables between genera clusters and both oxygen sensitivity and Gram stain status independently. To further investigate these links, we aimed to determine whether phenotypic characteristics could prelude the emergence of the storage bias that we observed. Therefore, for each solution, we performed a Kruskal-Wallis test among genera for log2-fold change and both oxygen sensitivity and Gram stain status independently.

## Results

In the present study, the performance of each stabilizer was defined as the microbial community alterations over time relative to the reference sample (i.e., immediately frozen sample stored with no additive). The technical reproducibility of our analytical protocol was evaluated using triplicates of reference samples, while samples with no additive (S and D) served as indicators of the natural evolution of the microbiota profile if unstabilized.

### Quality Control for DNA Yield, Purity and Alpha Diversity

**Figure 2:**
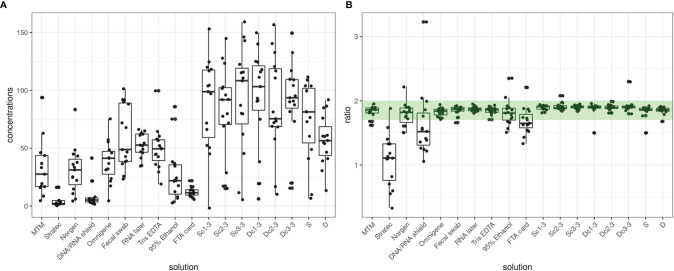
Quantity and quality of DNA extracted from human fecal samples. **(A)** DNA yield, expressed as ng/µl. **(B)** A 260/280 nm ratio indicative of the presence or absence of phenol, solvent and protein-type contaminants in the DNA extract. The green range indicates a ratio between 1.7 and 2.0, which here defines an optimal DNA quality.

Analysis of complex microbial ecosystems requires high-quality libraries for next generation sequencing (NGS) metagenomics. Hence, preserving a microbial profile over time and providing good DNA yield and purity of DNA extracts are key aspects in the analytical protocol in place. We found considerable differences in the DNA concentrations and A260/280 ratios of our extracted DNA. For example, Fecal Swab-preserved samples recovered, on average, 12-fold more DNA than Stratec-preserved samples (60.28 ng/µl vs 4.97 ng/µl). Among the different stabilizers tested in this study, recovered DNA was the lowest for Stratec, DNA/RNA Shield- and FTA card-stabilized samples (**Figure 2A**). In addition, samples preserved with these three solutions had primarily low A260/280 ratios (mean ratio <1.7), indicating the presence of contaminants (**Figure 2B**). Interestingly, Stratec samples were the least successful for recovering a microbiota profile with sufficient reads and showed a smaller alpha diversity than other stabilizers, while DNA/RNA Shield- and FTA card-preserved samples exhibited good profile recovery with high alpha diversity values (**Supplementary Figure S1**). The diversity index showed similar alpha diversity among preservation methods, and the Stratec stabilizer presented a much lower alpha diversity measure than the other stabilizers. As such, these results do not show any relationships among DNA concentration/purity, diversity and microbial profile recovery.

Dc and Sc measures resulted in similar concentrations and quality ratios among triplicate samples. Unstabilized samples (Dc, Sc, D and S) showed the highest DNA yield as they recovered on average 2.6-fold more DNA than stabilized samples with good A260/280 ratios.

A total of six samples were discarded, including five Stratec-stabilized samples and one Dc sample due to a lack of compliance with quality and/or quantity criteria.

### Homogenization of Stool Samples Results in Reduced Intrasample Variability

**Figure 3:**
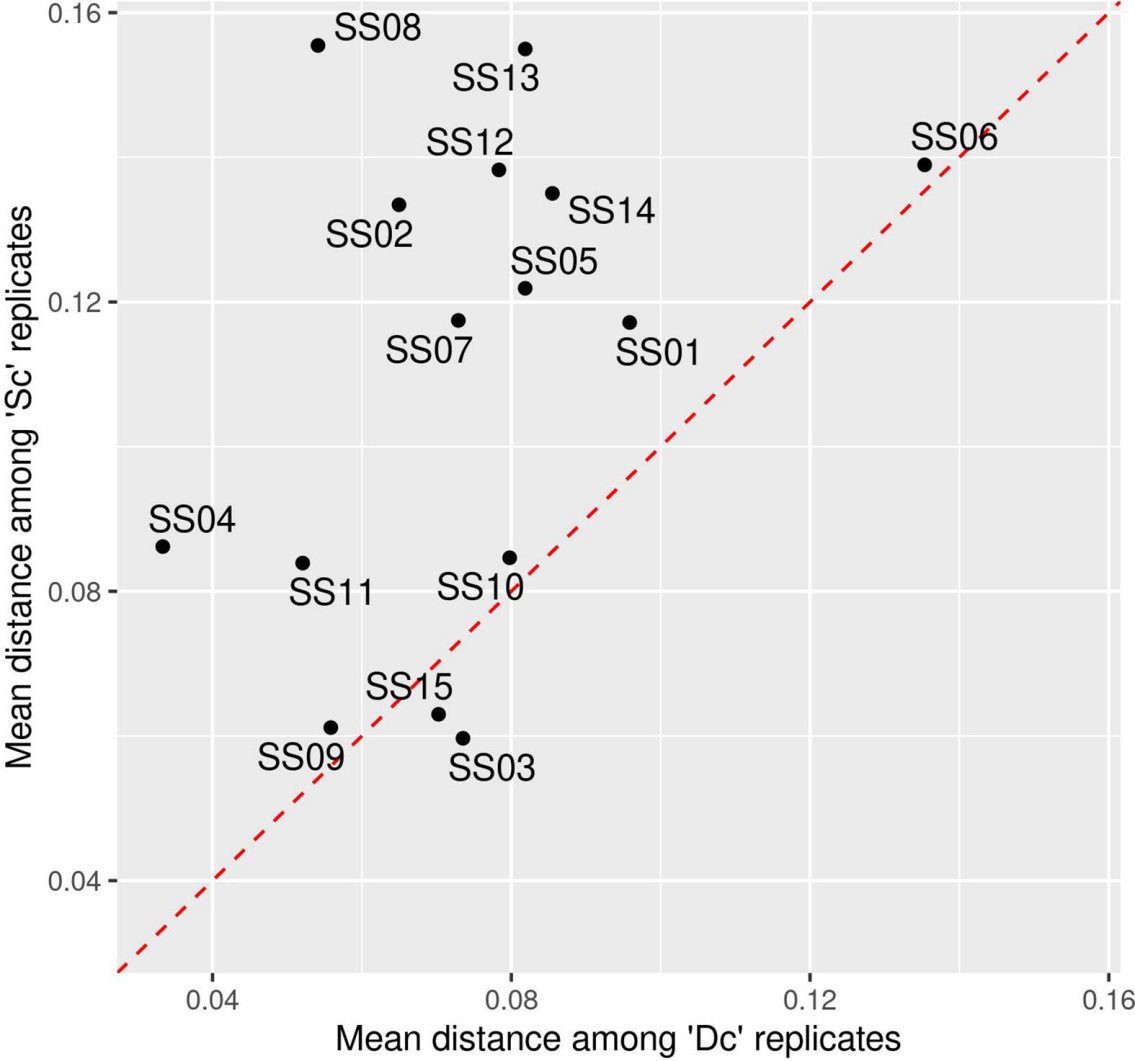
Mean dispersion using Bray-Curtis distances among technological replicates prior (Sc) and after (Dc) homogenization across 15 fecal samples (SS01 to SS15). The red dotted line indicates equality of dispersion among ‘Sc’ and ‘Dc’ samples.

Homogenization is commonly performed in studies to minimize intrasample variations and subsequent misestimation of the observed alterations within recovered profiles. The interaliquot variability for each Sc and Dc triplicate was first estimated by the mean distance using several methods (Bray-Curtis distance, Jaccard distance, and Aitchison distance). Comparison of distances within triplicates and between Sc and Dc triplicates showed a greater dispersion in Sc than in Dc triplicates, regardless of the distance method used (**Figure 3** and **Supplementary Figure S2**). In addition, a Wilcoxon test showed that homogenization significantly reduced observed interaliquot variability (*p=0.002*).

These results suggest that stool subsampling results in variations in the recovered microbial content among aliquots and confirms that homogenization of each sample has contributed here to significantly lowering the interaliquot variability. In this study, our homogenized aliquots added to the different stabilizers can thus be considered identical prior to storage. Their evolution over the 14-day storage period then provides an adequate evaluation of the efficacy of each stabilizer tested when compared to their reference (i.e., an average of Dc triplicates).

### DNA Stabilizers Alter Stool Microbial Composition With Various Orders of Magnitude Compared to Samples With No Additives

**Figure 4:**
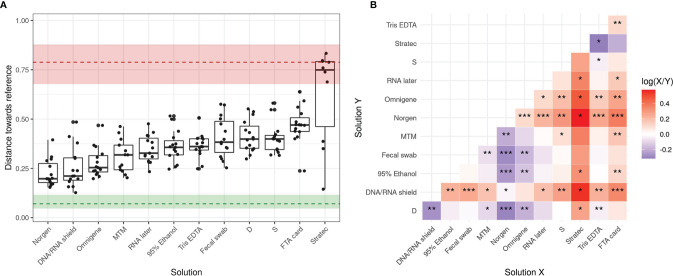
Summary of community shifts in response to stabilizing solutions over a 14-day storage period. **(A)** Bray-Curtis distance towards the reference for each patient grouped by stabilizing solution. Median and 5th-to-95th percentile ranges are shown for both interaliquot and interpatient variability. **(B)** A pairwise paired Wilcoxon test was performed to compare solutions with each other, the color code refers to the log10 fold change of the median performance across patients: blue if the solution on X axis performs better, red if the solution on Y axis performs better. Significance is shown as follows: (*) indicates fdr<0.05, (**) indicates fdr<0.01, (***) indicates fdr<0.001.

To evaluate the performance of the tested stabilizers, we quantified the compositional dissimilarity between each preserved sample and its reference, defined as the barycenter of the ‘Dc’ replicates. Different metrics, including the Bray-Curtis, Jaccard, and Aitchison distances, show that Norgen, DNA/RNA Shield, OMNIgene-Gut and PrimeStore MTM produced profiles closest to their reference, while the remaining stabilizers resulted in greater alterations (**Figure 4A** and **Supplementary Figures S3A**, **S4A**). A Kruskal-Wallis test (p<10^–11^) then confirmed that the solutions tested demonstrated distinct efficacies of stabilization specific to each stabilizer. Finally, a paired Wilcoxon test was used to compare the stabilizing performance among all stabilizers tested and identified Norgen as the best performing solution, closely followed by OMNIgene-Gut, DNA/RNA Shield and PrimeStore MTM, which presented similar performances (**Figure 4B** and **Supplementary Figures S3B**, **S4B**). In contrast, the least efficient stabilizers were Stratec, FTA card and Tris-EDTA, which appear no better than unstabilized samples (S or D).

In parallel, the results suggested that interindividual variability largely exceeded interaliquot variability (**Figure 4A** and **Supplementary Figures S3A**, **S4A**). Distances towards the reference were larger than interaliquot distances but smaller than those for interindividual variability indicating that preservation-induced effects were observed but were smaller than biological interindividual variability (**Supplementary Figure S5**). The only exception was Stratec-preserved samples, which displayed a variability similar to that observed among samples, confirming that this solution is not suitable for storage of human fecal samples. These results were confirmed by hierarchical clustering as shown in **Supplementary Figure S6**.

### Bacterial Relative Abundance Differs Based on the Method of Preservation in Different Taxonomic Ranks

**Figure 5:**
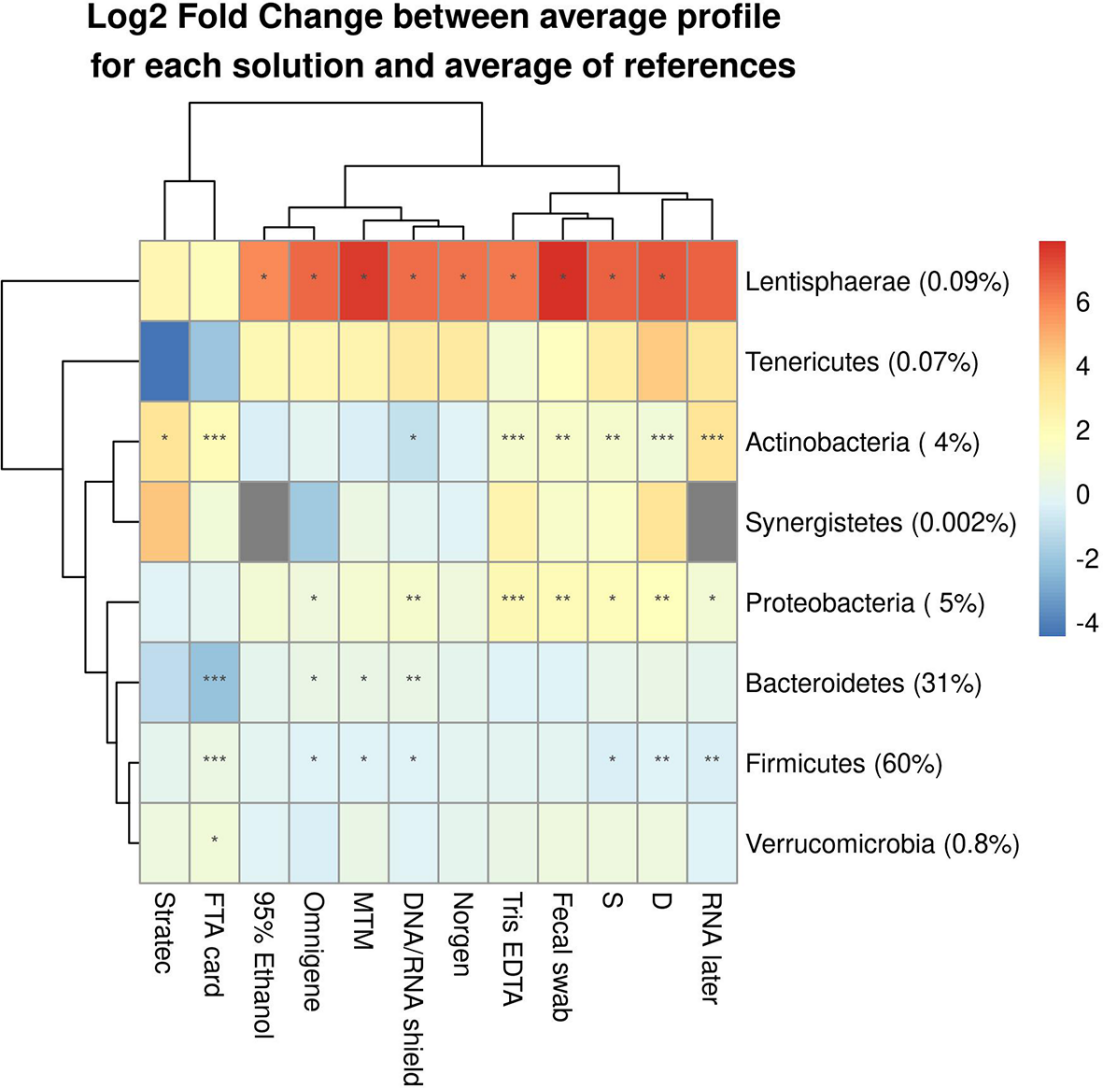
Differentially abundant bacterial phyla between samples and their references among the 10 tested DNA stabilizing solutions. The median log2-fold change of average profiles is shown with the significance according the corresponding paired Wilcoxon test. (*) indicates fdr<0.05, (**) indicates fdr<0.01, (***) indicates fdr<0.001.

Our analysis demonstrated that bacterial taxa were affected by the stabilizer, with misestimation of their relative abundance compared to their reference profiles (Dc). These alterations were detected at different taxonomic levels, including phyla (**Figure 5**) and genera (**Supplementary Figure S7**). Observed biases specific to each stabilizing solution were statistically confirmed by a paired Wilcoxon test, which showed that regardless of their efficiency of preserving a true microbiota profile, the different solutions tested impacted the relative abundance of certain bacterial taxa recovered when a fecal sample had been stored in a stabilizer. Low-abundance phyla (<1%), such as *Tenericutes*, *Synergistetes* and *Verrucomicrobia*, were the least significantly altered, except for *Lentisphaerae*, which was significantly overestimated in most storage conditions tested. Among abundant phyla (>1%), the most significantly affected were *Actinobacteria* and *Proteobacteria*, which tended to be overestimated, while *Firmicutes* and *Bacteroidetes* were underestimated. Of all abundant phyla, *Bacteroidetes* were interestingly the least significantly altered.

Parallel samples that were not exposed to any additive (S, D) also showed profile alterations, suggesting an effect of storage temporality, likely due to both bacterial growth for some populations and bacterial death for others. The lack of stabilization at fluctuating temperatures resulted in significant alterations of *Firmicutes*, *Proteobacteria*, *Actinobacteria* and *Lentisphaerae*.

Among the solutions with the greatest performances for stabilizing the fecal microbiota, Norgen did not significantly alter any phyla, except for *Lentisphaerae*, which were overestimated. In contrast, significant alterations were observed with OMNIgene-Gut and DNA/RNA Shield, both of which significantly affected *Firmicutes*, *Bacteroidetes*, *Proteobacteria* and *Lentisphaerae*, while *Actinobacteria* was only affected by DNA/RNA Shield. Interestingly, PrimeStore MTM appeared to significantly disturb only *Firmicutes* and *Lentisphaerae*. Stratec, which was the least efficient for preserving the fecal microbiota in our study, seemed to only affect *Actinobacteria*, but this result is biased, as many Stratec-preserved samples were excluded from this analysis due to poor quality DNA and/or low read numbers compared to the other stabilizing methods.

Considering the diversity of populations that can be found within phyla and the possibility that some phenotypic characteristics may dictate or facilitate certain alterations, it is interesting to observe changes at a lower taxonomic range. Genera clusters, based on alterations in the microbiota profile across all solutions tested, were found to be dependent on both oxygen sensitivity (p=0.0002) and Gram stain status (p=0.049) among the genera retrieved (**Supplementary Figure S7** and **Supplementary Table ST1**). These results indicate that the physiological traits examined could potentially prelude which populations are susceptible to alteration by the DNA stabilizers. For each solution, we tested the effect of these phenotypical characteristics on the log2 fold change between a stabilized sample and its reference. We found no significant effect of oxygen sensitivity or Gram stain status on genus alterations in the absence of stabilizing solutions (samples S and D). In contrast, for stabilized samples, we found that genus alteration during storage was influenced by their oxygen status for Tris-EDTA (p=0.025) and in FTA card (p=0.029). Similarly, we found that Gram stain status affected samples stabilized with DNA/RNA Shield (p=0.002), PrimeStore MTM (p=0.027) and Stratec (p=0.043).

## Discussion

To the best of our knowledge, this study is the first in the microbiome field to compare such a large panel of storage methods, allowing identification of the best performing DNA stabilizers for a given ecosystem. We have shown that, of all stabilizers tested, some drastically impact the observed microbial composition and introduce biases. To proceed, we chose to evaluate methodologies already in use in the microbiome field through a comparative study of 10 storage methods to identify optimal fecal sampling methods that provide reproducible, stable, and accurate results.

Our analysis identified Norgen, OMNIgene-Gut, DNA/RNA Shield and PrimeStore MTM as the most efficacious stabilizers as compared to the immediately frozen aliquots (Dc). According to our results, several comparative studies have identified OMNIgene-Gut as a good DNA stabilizer for microbiome studies (Choo et al., 2015; Song et al., 2016; Abrahamson et al., 2017; Williams et al., 2019), while the other three solutions have not yet been extensively evaluated by comparative studies. In contrast, the remaining solutions tested were less efficient, showing a profile with alterations similar to unstabilized samples (S and D). Interestingly, among the stabilizers that were less reliable in our analysis, most showed discordant results in their ability to preserve fecal samples throughout comparative studies. For example, RNAlater was until recently the most commonly used buffer for metagenomic studies (Nechvatal et al., 2008; Cardona et al., 2012; Dominianni et al., 2014; Choo et al., 2015; Flores et al., 2015; Thomas et al., 2015; Song et al., 2016). However, its suitability for microbiome analysis has been extensively reviewed, as some studies claim that it results in reduced overall DNA yields and reduces the detection/abundance of bacterial taxa (Dominianni et al., 2014; Choo et al., 2015; Gorzelak et al., 2015; Hale et al., 2015; Sinha et al., 2016; Hickl et al., 2019). Our results did not show reduced DNA yield compared to other preserved solutions but did show significant alterations in the recovered microbiota compared to their references, thus agreeing with previous studies that RNAlater is not an optimal preservation method. We came to the same conclusion for FTA card as Hale et al. (Hale et al., 2015), who demonstrated that FTA card (and RNAlater)-preserved samples were the least similar to fresh samples, while in contrast, Sinha et al. (Sinha et al., 2016) recommended the use of FTA card for short-term storage, demonstrating that it provides reproducible, stable, and accurate data across laboratories (over 4-day storage). The longer storage time in our protocol might have contributed to our discordant results. Similar to numerous studies performing homogenization of fecal samples (Carroll et al., 2012; Choo et al., 2015; Sinha et al., 2016; Song et al., 2016; Shaw et al., 2016; Vogtmann et al., 2017a; Vogtmann et al., 2017b), homogenization of our samples contributed to a better evaluation of the true performance of each stabilizing solution for preserving the microbiota content over time, as each aliquot presented a similar profile when added to the stabilizer.

Despite various effective methods for preserving a true microbiota profile over storage time, the alterations observed between the reference samples and their 14-day-stabilized aliquots were smaller than the differences between samples (subjects), except for Stratec-preserved samples. Furthermore, triplicates for each stool sample collected did not cluster by preservation method. Therefore, the human gut appears to be highly subject-specific, as our results suggest that interindividual variation accounts for the major of differences observed in fecal samples and outweighs the effect (or bias) of collection and storage, as previously demonstrated in several studies (Choo et al., 2015; Voigt et al., 2015; Guo et al., 2016; Sinha et al., 2016; Song et al., 2016). As stated above, the only exception was Stratec-preserved samples, which displayed variability similar to that observed among samples, indicating that this solution is not suitable for storage of human fecal samples. This result contradicts a recent study (Chen et al., 2020), which concluded that the Stratec solution was a suitable storage buffer for fecal specimen preservation. However, Chen et al. performed this study on a small cohort (n=4) over a 7-day period of storage at room temperature. The fluctuating temperatures in our protocol and the longer period of storage might explain the discrepancies between these findings. Additionally, our results did not demonstrate any relationships among DNA concentration/purity, microbial diversity, and microbial composition, similar to previous studies (Salonen et al., 2010; Hale et al., 2015). However, it has been suggested that high DNA concentrations might favor the identification of rare populations (Dominianni et al., 2014; Choo et al., 2015; Gorzelak et al., 2015; Hale et al., 2015). Although the low DNA yield observed with Stratec-stabilized samples might not entirely explain the difficulties in recovering a good microbiota profile, this factor may have contributed to its poor performances in our protocol.

Finally, microbiome comparative studies investigating the effect of storage often examine variations in the relative abundances of phyla and genera specific to the stabilizing methods. However, they do not examine these alterations based on microbial population characteristics, with the literature showing that bacteria within a genus share the same general phenotypic characteristics, in particular oxygen sensitivity and Gram stain status (Schmaljohn and McClain, 1996; Brenner et al., 2005; Lowy, 2009). In this study, we demonstrated that altered dynamics resulting from sample preservation are dictated by the phenotypical characteristics of the bacterial populations present in the studied sample. Our samples showed that genera alteration during storage is influenced by oxygen status for the Tris-EDTA and FTA card methods, as well as the Gram stain status for the DNA/RNA Shield, PrimeStore MTM and Stratec methods. A recent study also demonstrated that Gram status can alter the microbial content when Norgen stabilizer is used (Watson et al., 2019). Hence, preservation of the microbiota profile is impacted by the stabilizer chosen and its efficacy for preserving the true microbial profile. However, it must be taken into consideration that the stabilizer’s performance can also be affected by the microbial content of the studied sample and its most common phenotypical traits.

One limitation of our study is that we did not evaluate the stabilizing performances of each solution tested across different times or over long-term storage periods. Indeed, Sinha et al. (Sinha et al., 2016) found that incubation at room temperature over 4 days reduced the reproducibility for most sampling methods, including no additives, swab, 70% ethanol, and EDTA. As such, the performance measures in our study only reflect their efficacies over a period of 14 days throughout various temperature fluctuations but do not attest of the loss of technical reproducibility or the impact on the alteration of bacterial taxa if samples are incubated in their stabilizers for a longer period. Considering we demonstrate here that genera alteration is influenced by oxygen status, our results may therefore underestimate the impact of the stabilizing solution on oxygen-sensitive bacterial population for the samples collected at home. Finally, our results here show that the choice of the stabilizer could be impacted by the microbial composition and phenotypic traits of the studied samples, but our study only analyzed the human gut ecosystem of a French cohort on a small group of individuals. Hence efficacies of the different DNA stabilizers used by the scientific community could vary depending on the microbial composition of the studied population. A study of a larger cohort with varying genetics (from different ethnic groups) and environmental factors could then lead to different conclusions and rank the efficacy of the DNA stabilizers differently. Further studies will be required in order to provide the scientific community with a more comprehensive analysis of stabilizing methods throughout different cohorts and with different types of samples to establish guidelines that will help scientists in their experimental settings.

We anticipate that procedures for microbial preservation will likely further improve in the future, and we show with this study that preservation remains a key step that can introduce technical bias into the study of complex ecosystems such as the human gut. Here, we demonstrated that some stabilizers are not suitable for the preservation of a stool sample when the sample is intended to describe the whole complexity of the human gut ecosystem through 16S metagenomics. Our data identified Norgen, OMNIgene-Gut, DNA/RNA Shield and PrimeStore MTM as the most effective stabilizers, as they resulted in reduced technical biases. Acknowledging the performances of stabilizing solutions and their suitability depending on the microbial content of the ecosystem studied will help establish standards in omics studies. If implemented within metagenomics protocols across laboratories, these solutions could promote experimental reproducibility among research groups and lead to meaningful knowledge about the gut microbiome and its impact on human health with the discovery of new health-associated microbiome patterns and biomarkers.

## Conclusion

The diversity and complexity of the human gut microbiota increase the difficulty of elaborating a method to study such ecosystems without experimental biases. Storage conditions can introduce substantial changes to microbial community profiling in regard to 16S metagenomics. Acknowledging the biases and limitations of the implemented method is key to better interpret and support true health (disease)-associated microbiome patterns that will then lead us towards personalized medicine, in which the microbiota profile could constitute a reliable tool for clinical practice.

## Data Availability Statement

The data for this study have been deposited in the European Nucleotide Archive (ENA) at EMBL-EBI under accession number PRJEB40569 (https://www.ebi.ac.uk/ena/browser/view/PRJEB40569). Scripts are available at https://gitcrcm.marseille.inserm.fr/goutorbe/stool-preservation.

## Ethics Statement

Ethical review and approval was not required for the study on human participants in accordance with the local legislation and institutional requirements. The patients/participants provided their written informed consent to participate in this study.

## Author Contributions

AP designed the study, extracted and sequenced samples, analyzed data, and wrote and finalized the manuscript. ET extracted and sequenced samples and drafted the manuscript. BG analyzed data and drafted the manuscript. MB extracted and sequenced samples. GP analyzed data. PH finalized the manuscript and funded this study. All authors discussed the results and commented on the manuscript. All authors contributed to the article and approved the submitted version.

## Funding

This research received no specific grant from any funding agency in the public, commercial, or not-for-profit sectors. Alphabio laboratory funded this study.

## Conflict of Interest

The authors declare that the research was conducted in the absence of any commercial or financial relationships that could be construed as a potential conflict of interest.

## Publisher’s Note

All claims expressed in this article are solely those of the authors and do not necessarily represent those of their affiliated organizations, or those of the publisher, the editors and the reviewers. Any product that may be evaluated in this article, or claim that may be made by its manufacturer, is not guaranteed or endorsed by the publisher.

## Supplementary Material

Supplementary Figure 1 Effects of storage conditions on alpha diversity with respect to the observed richness and Shannon index.

Supplementary Figure 2 Mean dispersion using the Jaccard distance **(A)** or Aitchison distance **(B)** among technological replicates prior to (Sc) and after (Dc) homogenization across 15 fecal samples. The red dotted lines indicate the equality of dispersion among ‘Sc’ and ‘Dc’ samples.

Supplementary Figure 3 Summary of community shifts in response to stabilizing solutions over a 14-day storage period. **(A)** Jaccard distance towards the reference for each participant, grouped by stabilizing solution, and the median and 5th-to-95th percentile range are shown for both interaliquot and interpatient variability. **(B)** A pairwise paired Wilcoxon test was performed to compare solutions with each other, the color code refers to the log10 fold change of the median performance across patients: blue means that the solution on X axis performs better, red means that the solution on Y axis performs better. Significance is shown as follows: (*) indicates fdr<0.05, (**) indicates fdr<0.01, (***) indicates fdr<0.001.

Supplementary Figure 4 Summary of community shifts in response to stabilizing solutions over a 14-day storage period. **(A)** The Aitchison distance towards the reference for each participant, grouped by stabilizing solution, median and 5th-to-95th percentile range are shown for both interaliquot and interpatient variability. **(B)** A pairwise paired Wilcoxon test was performed to compare solutions with each other, the color code refers to the log10 fold change of the median performance across patients: blue means that the solution on X axis performs better, red means that the solution on Y axis performs better. Significance is shown as follows: (*) indicates fdr<0.05, (**) indicates fdr<0.01, (***) indicates fdr<0.001.

Supplementary Figure 5 Principal coordinate analysis (PCoA) based on Bray-Curtis distances computed from the rarefied data set. The plots are split according to samples origins, colored and labelled according to storage method. Points labelled as REF refers to the reference used for evaluation of storage methods for each patient, defined as the mean of the Dc samples.

Supplementary Figure 6 Hierarchical clustering based on the Bray-Curtis distance matrix of all samples in the data set. The first 4 digits of the sample IDs refer to the biological origin of the fecal sample, and the remaining digits refer to the storage conditions (i.e., Sc, Dc, D, S or stabilizing solutions). Technological replicates clustered together, except for *SS01Sc1-3*, *SS02Sc1-3* and *SS04Dc3-3* (shown in red), which were excluded from downstream analysis.

Supplementary Figure 7 Differentially abundant bacterial genera among samples and their references among 10 tested DNA stabilizing solutions. The median log2-fold change between average profiles and significance of the corresponding paired Wilcoxon test are shown. (*) indicates fdr<0.05, (**) indicates fdr<0.01, (***) indicates fdr<0.001.

Supplementary Table 1 Phenotypic characteristics of the 50 most abundant genera recovered following a 14-day storage period. NEG, negative Gram strain; POS, positive Gram strain; VAR, variable Gram stain; ANAER-ST, strict anaerobic; AER-ST, strict aerobic; FAC, facultative anaerobic; MICRO-AE, microaerophile.* Proportion (%), average relative abundance of each genera within the overall dataset.

## References

[B1] Abrahamson M. Hooker E. Ajami N. J. Petrosino J. F. Orwoll E. S. (2017). Successful Collection of Stool Samples for Microbiome Analyses From a Large Community-Based Population of Elderly Men. Contemp. Clin. Trials Commun. 7, 158–162. doi: 10.1016/j.conctc.2017.07.002 29250605 PMC5726580

[B2] Bahl M. I. Bergström A. Licht T. R. (2012). Freezing Fecal Samples Prior to DNA Extraction Affects the Firmicutes to Bacteroidetes Ratio Determined by Downstream Quantitative PCR Analysis. FEMS Microbiol. Lett. 329, 193–197. doi: 10.1111/j.1574-6968.2012.02523.x 22325006

[B3] Bolyen E. Rideout J. R. Dillon M. R. Bokulich N. A. Abnet C. C. Al-Ghalith G. A.. (2019). Reproducible, Interactive, Scalable and Extensible Microbiome Data Science Using QIIME 2. Nat. Biotechnol. 37, 852–857. doi: 10.1038/s41587-019-0209-9 31341288 PMC7015180

[B4] Brenner D. J. Staley J. T. Krieg N. R. (2005). “Classification of Procaryotic Organisms and the Concept of Bacterial Speciation,” in Bergey’s Manual® of Systematic Bacteriology: Volume Two: The Proteobacteria, Part A Introductory Essays. Eds. Brenner D. J. Krieg N. R. Staley J. T. Garrity G. M. (Boston, MA: Springer US), 27–32. doi: 10.1007/0-387-28021-9_4

[B5] Callahan B. J. McMurdie P. J. Rosen M. J. Han A. W. Johnson A. J. A. Holmes S. P. (2016). DADA2: High-Resolution Sample Inference From Illumina Amplicon Data. Nat. Methods 13, 581–583. doi: 10.1038/nmeth.3869 27214047 PMC4927377

[B6] Cardona S. Eck A. Cassellas M. Gallart M. Alastrue C. Dore J.. (2012). Storage Conditions of Intestinal Microbiota Matter in Metagenomic Analysis. BMC Microbiol. 12, 1–8. doi: 10.1186/1471-2180-12-158 22846661 PMC3489833

[B7] Carroll I. M. Ringel-Kulka T. Siddle J. P. Klaenhammer T. R. Ringel Y. (2012). Characterization of the Fecal Microbiota Using High-Throughput Sequencing Reveals a Stable Microbial Community During Storage. PloS One 7, e46953. doi: 10.1371/journal.pone.0046953 23071673 PMC3465312

[B8] Chen C.-C. Wu W.-K. Chang C.-M. Panyod S. Lu T.-P. Liou J.-M.. (2020). Comparison of DNA Stabilizers and Storage Conditions on Preserving Fecal Microbiota Profiles. J. Formosan. Med. Assoc. 119 (12), 1791–1798. doi: 10.1016/j.jfma.2020.01.013 32111519

[B9] Choo J. M. Leong L. E. Rogers G. B. (2015). Sample Storage Conditions Significantly Influence Faecal Microbiome Profiles. Sci. Rep. 5, 1–10. doi: 10.1038/srep16350 PMC4648095 26572876

[B10] Conrads G. Abdelbary M. M. (2019). Challenges of Next-Generation Sequencing Targeting Anaerobes. Anaerobe 58, 47–52. doi: 10.1016/j.anaerobe.2019.02.006 30769104

[B11] Costea P. I. Zeller G. Sunagawa S. Pelletier E. Alberti A. Levenez F.. (2017). Towards Standards for Human Fecal Sample Processing in Metagenomic Studies. Nat. Biotechnol. 35, 1069–1076. doi: 10.1038/nbt.3960 28967887

[B12] Cryan J. F. Dinan T. G. (2012). Mind-Altering Microorganisms: The Impact of the Gut Microbiota on Brain and Behaviour. Nat. Rev. Neurosci. 13, 701–712. doi: 10.1038/nrn3346 22968153

[B13] Dominianni C. Wu J. Hayes R. B. Ahn J. (2014). Comparison of Methods for Fecal Microbiome Biospecimen Collection. BMC Microbiol. 14, 103. doi: 10.1186/1471-2180-14-103 24758293 PMC4005852

[B14] Flores R. Shi J. Yu G. Ma B. Ravel J. Goedert J. J.. (2015). Collection Media and Delayed Freezing Effects on Microbial Composition of Human Stool. Microbiome 3, 33. doi: 10.1186/s40168-015-0092-7 26269741 PMC4534027

[B15] Fouhy F. Deane J. Rea M. C. O’Sullivan Ó. Ross R. P. O’Callaghan G.. (2015). The Effects of Freezing on Faecal Microbiota as Determined Using MiSeq Sequencing and Culture-Based Investigations. PloS One 10, e0119355. doi: 10.1371/journal.pone.0119355 25748176 PMC4352061

[B16] Gloor G. B. Macklaim J. M. Pawlowsky-Glahn V. Egozcue J. J. (2017). Microbiome Datasets Are Compositional: And This Is Not Optional. Front. Microbiol. 8. doi: 10.3389/fmicb.2017.02224 PMC5695134 29187837

[B17] Gorzelak M. A. Gill S. K. Tasnim N. Ahmadi-Vand Z. Jay M. Gibson D. L. (2015). Methods for Improving Human Gut Microbiome Data by Reducing Variability Through Sample Processing and Storage of Stool. PloS One 10, e0134802. doi: 10.1371/journal.pone.0134802 26252519 PMC4529225

[B18] Guo Y. Li S.-H. Kuang Y.-S. He J.-R. Lu J.-H. Luo B.-J.. (2016). Effect of Short-Term Room Temperature Storage on the Microbial Community in Infant Fecal Samples. Sci. Rep. 6, 26648. doi: 10.1038/srep26648 27226242 PMC4880902

[B19] Hale V. L. Tan C. L. Knight R. Amato K. R. (2015). Effect of Preservation Method on Spider Monkey (Ateles Geoffroyi) Fecal Microbiota Over 8weeks. J. Microbiol. Methods 113, 16–26. doi: 10.1016/j.mimet.2015.03.021 25819008

[B20] Hickl O. Heintz-Buschart A. Trautwein-Schult A. Hercog R. Bork P. Wilmes P.. (2019). Sample Preservation and Storage Significantly Impact Taxonomic and Functional Profiles in Metaproteomics Studies of the Human Gut Microbiome. Microorganisms 7, 367. doi: 10.3390/microorganisms7090367 PMC6780314 31546776

[B21] Hornung B. V. Zwittink R. D. Kuijper E. J. (2019). Issues and Current Standards of Controls in Microbiome Research. FEMS Microbiol. Ecol. 95, fiz045. doi: 10.1093/femsec/fiz045 30997495 PMC6469980

[B22] Illumina (2013) 16s Sample Preparation Guide. Available at: https://support.illumina.com/documents/documentation/chemistry_documentation/16s/16s-metagenomic-library-prep-guide-15044223-b.pdf (Accessed June 7, 2021).

[B23] Kim D. Hofstaedter C. E. Zhao C. Mattei L. Tanes C. Clarke E.. (2017). Optimizing Methods and Dodging Pitfalls in Microbiome Research. Microbiome 5, 1–14. doi: 10.1186/s40168-017-0267-5 28476139 PMC5420141

[B24] Lauber C. L. Zhou N. Gordon J. I. Knight R. Fierer N. (2010). Effect of Storage Conditions on the Assessment of Bacterial Community Structure in Soil and Human-Associated Samples. FEMS Microbiol. Lett. 307, 80–86. doi: 10.1111/j.1574-6968.2010.01965.x 20412303 PMC3148093

[B25] Ley R. E. Turnbaugh P. J. Klein S. Gordon J. I. (2006). Human Gut Microbes Associated With Obesity. Nature 444, 1022–1023. doi: 10.1038/4441022a 17183309

[B26] Louis P. Hold G. L. Flint H. J. (2014). The Gut Microbiota, Bacterial Metabolites and Colorectal Cancer. Nat. Rev. Microbiol. 12, 661–672. doi: 10.1038/nrmicro3344 25198138

[B27] Lowy F. (2009). Bacterial Classification, Structure and Function (New York, USA: Columbia University), 1–6.

[B28] Lozupone C. A. Stombaugh J. I. Gordon J. I. Jansson J. K. Knight R. (2012). Diversity, Stability and Resilience of the Human Gut Microbiota. Nature 489, 220–230. doi: 10.1038/nature11550 22972295 PMC3577372

[B29] Lu J. Salzberg S. L. (2020). Ultrafast and Accurate 16S rRNA Microbial Community Analysis Using Kraken 2. Microbiome 8, 124. doi: 10.1186/s40168-020-00900-2 32859275 PMC7455996

[B30] McMurdie P. J. Holmes S. (2013). Phyloseq: An R Package for Reproducible Interactive Analysis and Graphics of Microbiome Census Data. PloS One 8, e61217. doi: 10.1371/journal.pone.0061217 23630581 PMC3632530

[B31] Nechvatal J. M. Ram J. L. Basson M. D. Namprachan P. Niec S. R. Badsha K. Z.. (2008). Fecal Collection, Ambient Preservation, and DNA Extraction for PCR Amplification of Bacterial and Human Markers From Human Feces. J. Microbiol. Methods 72, 124–132. doi: 10.1016/j.mimet.2007.11.007 18162191

[B32] Nguyen T. L. A. Vieira-Silva S. Liston A. Raes J. (2015). How Informative is the Mouse for Human Gut Microbiota Research? Dis. Models Mech. 8, 1–16. doi: 10.1242/dmm.017400 PMC4283646 25561744

[B33] Parte A. C. (2018). LPSN - List of Prokaryotic Names With Standing in Nomenclature (Bacterio.Net), 20 Years on. Int. J. Syst. Evol. Microbiol. 68, 1825–1829. doi: 10.1099/ijsem.0.002786 29724269

[B34] Roesch L. F. Casella G. Simell O. Krischer J. Wasserfall C. H. Schatz D.. (2009). Influence of Fecal Sample Storage on Bacterial Community Diversity. Open Microbiol. J. 3, 40. doi: 10.2174/1874285800903010040 19440250 PMC2681173

[B35] Salonen A. Nikkilä J. Jalanka-Tuovinen J. Immonen O. Rajilić-Stojanović M. Kekkonen R. A.. (2010). Comparative Analysis of Fecal DNA Extraction Methods With Phylogenetic Microarray: Effective Recovery of Bacterial and Archaeal DNA Using Mechanical Cell Lysis. J. Microbiol. Methods 81, 127–134. doi: 10.1016/j.mimet.2010.02.007 20171997

[B36] Sartor R. B. (2008). Microbial Influences in Inflammatory Bowel Diseases. Gastroenterology 134, 577–594. doi: 10.1053/j.gastro.2007.11.059 18242222

[B37] Schmaljohn A. L. McClain D. (1996). “Alphaviruses (Togaviridae) and Flaviviruses (Flaviviridae),” in Medical Microbiology, 4th Gavelston (University of Texas Medical Branch at Galveston). 21413253

[B38] Sekirov I. Russell S. L. Antunes L. C. M. Finlay B. B. (2010). Gut Microbiota in Health and Disease. Physiol. Rev. 90, 859–904. doi: 10.1152/physrev.00045.2009 20664075

[B39] Shaw A. G. Sim K. Powell E. Cornwell E. Cramer T. McClure Z. E.. (2016). Latitude in Sample Handling and Storage for Infant Faecal Microbiota Studies: The Elephant in the Room? Microbiome 4, 40. doi: 10.1186/s40168-016-0186-x 27473284 PMC4967342

[B40] Sinha R. Chen J. Amir A. Vogtmann E. Shi J. Inman K. S.. (2016). Collecting Fecal Samples for Microbiome Analyses in Epidemiology Studies. Cancer Epidemiol. Biomarkers Prev. 25, 407–416. doi: 10.1158/1055-9965.EPI-15-0951 26604270 PMC4821594

[B41] Sommer F. Bäckhed F. (2013). The Gut Microbiota—Masters of Host Development and Physiology. Nat. Rev. Microbiol. 11, 227–238. doi: 10.1038/nrmicro2974 23435359

[B42] Song S. J. Amir A. Metcalf J. L. Amato K. R. Xu Z. Z. Humphrey G.. (2016). Preservation Methods Differ in Fecal Microbiome Stability, Affecting Suitability for Field Studies. mSystems 1, e00021–16. doi: 10.1128/mSystems.00021-16 PMC5069758 27822526

[B43] Thomas V. Clark J. Doré J. (2015), e00021–16. Fecal Microbiota Analysis: An Overview of Sample Collection Methods and Sequencing Strategies. Future Microbiol. 10, 1485–1504. doi: 10.2217/fmb.15.87 26347019

[B44] Vogtmann E. Chen J. Amir A. Shi J. Abnet C. C. Nelson H.. (2017a). Comparison of Collection Methods for Fecal Samples in Microbiome Studies. Am. J. Epidemiol. 185, 115–123. doi: 10.1093/aje/kww177 27986704 PMC5253972

[B45] Vogtmann E. Chen J. Kibriya M. G. Chen Y. Islam T. Eunes M.. (2017b). Comparison of Fecal Collection Methods for Microbiota Studies in Bangladesh. Appl. Environ. Microbiol. 83, e00361–17. doi: 10.1128/AEM.00361-17 28258145 PMC5411505

[B46] Voigt A. Y. Costea P. I. Kultima J. R. Li S. S. Zeller G. Sunagawa S.. (2015). Temporal and Technical Variability of Human Gut Metagenomes. Genome Biol. 16, 73. doi: 10.1186/s13059-015-0639-8 25888008 PMC4416267

[B47] Watson E.-J. Giles J. Scherer B. L. Blatchford P. (2019). Human Faecal Collection Methods Demonstrate a Bias in Microbiome Composition by Cell Wall Structure. Sci. Rep. 9, 1–18. doi: 10.1038/s41598-019-53183-5 31727963 PMC6856092

[B48] Wen L. Ley R. E. Volchkov P. Y. Stranges P. B. Avanesyan L. Stonebraker A. C.. (2008). Innate Immunity and Intestinal Microbiota in the Development of Type 1 Diabetes. Nature 455, 1109–1113. doi: 10.1038/nature07336 18806780 PMC2574766

[B49] Williams G. M. Leary S. D. Ajami N. J. Chipper Keating S. Petrosin J. F. Hamilton-Shield J. P.. (2019). Gut Microbiome Analysis by Post: Evaluation of the Optimal Method to Collect Stool Samples From Infants Within a National Cohort Study. PloS One 14, e0216557. doi: 10.1371/journal.pone.0216557 31188837 PMC6561628

[B50] Wood D. E. Salzberg S. L. (2014). Kraken: Ultrafast Metagenomic Sequence Classification Using Exact Alignments. Genome Biol. 15, R46. doi: 10.1186/gb-2014-15-3-r46 24580807 PMC4053813

[B51] Wu W.-K. Chen C.-C. Panyod S. Chen R.-A. Wu M.-S. Sheen L.-Y.. (2019). Optimization of Fecal Sample Processing for Microbiome Study—The Journey From Bathroom to Bench. J. Formosan. Med. Assoc. 118, 545–555. doi: 10.1016/j.jfma.2018.02.005 29490879

[B52] Wu G. D. Lewis J. D. Hoffmann C. Chen Y.-Y. Knight R. Bittinger K.. (2010). Sampling and Pyrosequencing Methods for Characterizing Bacterial Communities in the Human Gut Using 16S Sequence Tags. BMC Microbiol. 10, 206. doi: 10.1186/1471-2180-10-206 20673359 PMC2921404

