# Supplementary figures and images for: Human Stool Preservation Impacts Taxonomic Profiles in 16S Metagenomics Studies
Source: Front Cell Infect Microbiol. 2022 Feb 8;12:722886. doi: 10.3389/fcimb.2022.722886 (PMC8860989; doi:10.3389/fcimb.2022.722886)

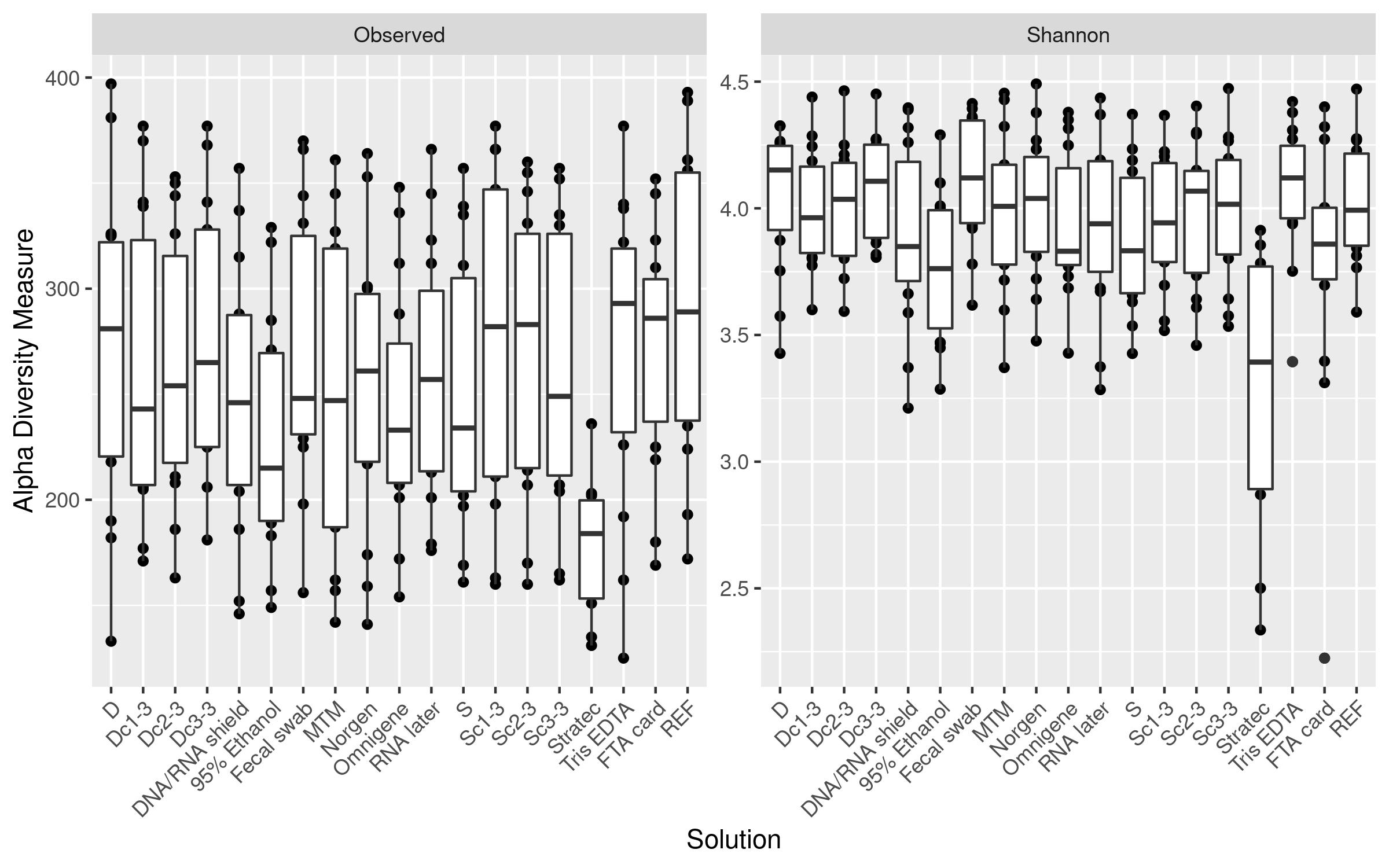

Supplement: Supplementary Figure 1 — Effects of storage conditions on alpha diversity with respect to the observed richness and Shannon index. [file Image_1.jpeg]

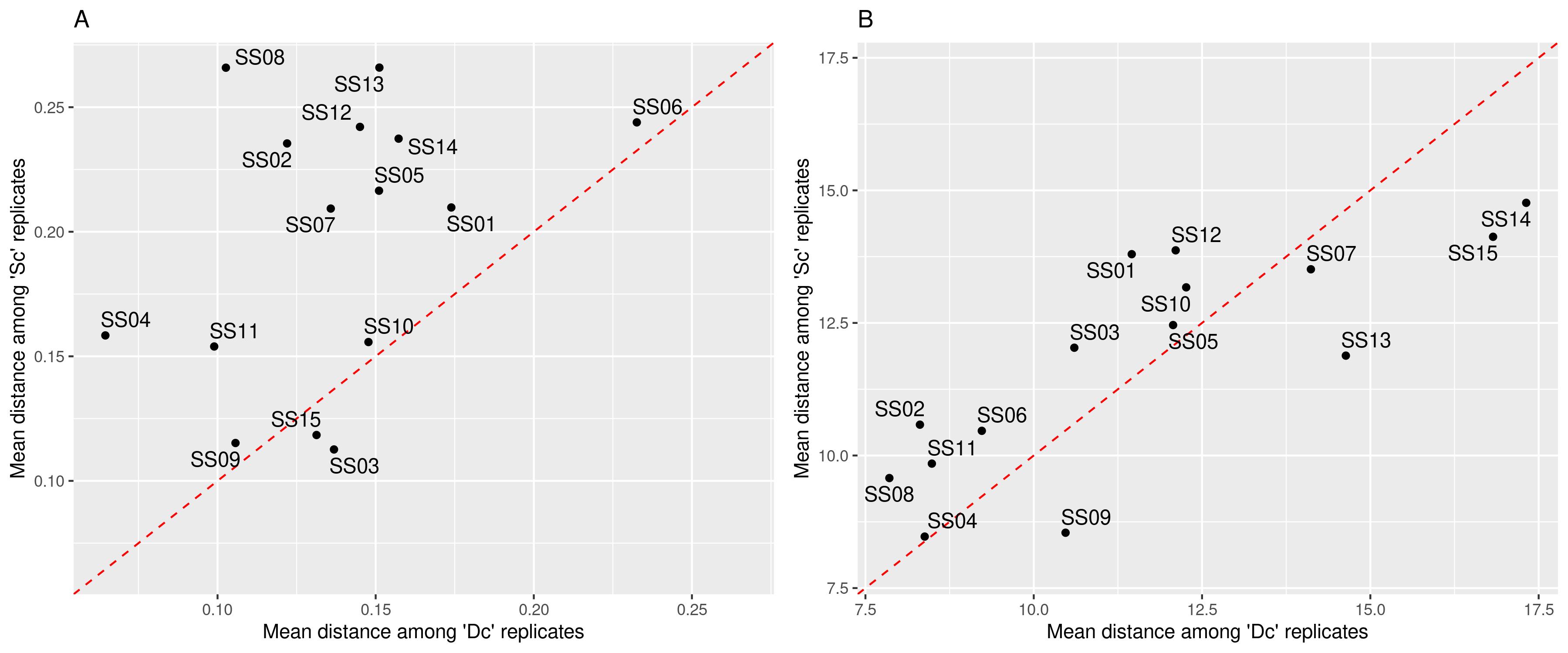

Supplement: Supplementary Figure 2 — Mean dispersion using the Jaccard distance (A) or Aitchison distance (B) among technological replicates prior to (Sc) and after (Dc) homogenization across 15 fecal samples. The red dotted lines indicate the equality of dispersion among ‘Sc’ and ‘Dc’ samples. [file Image_2.jpeg]

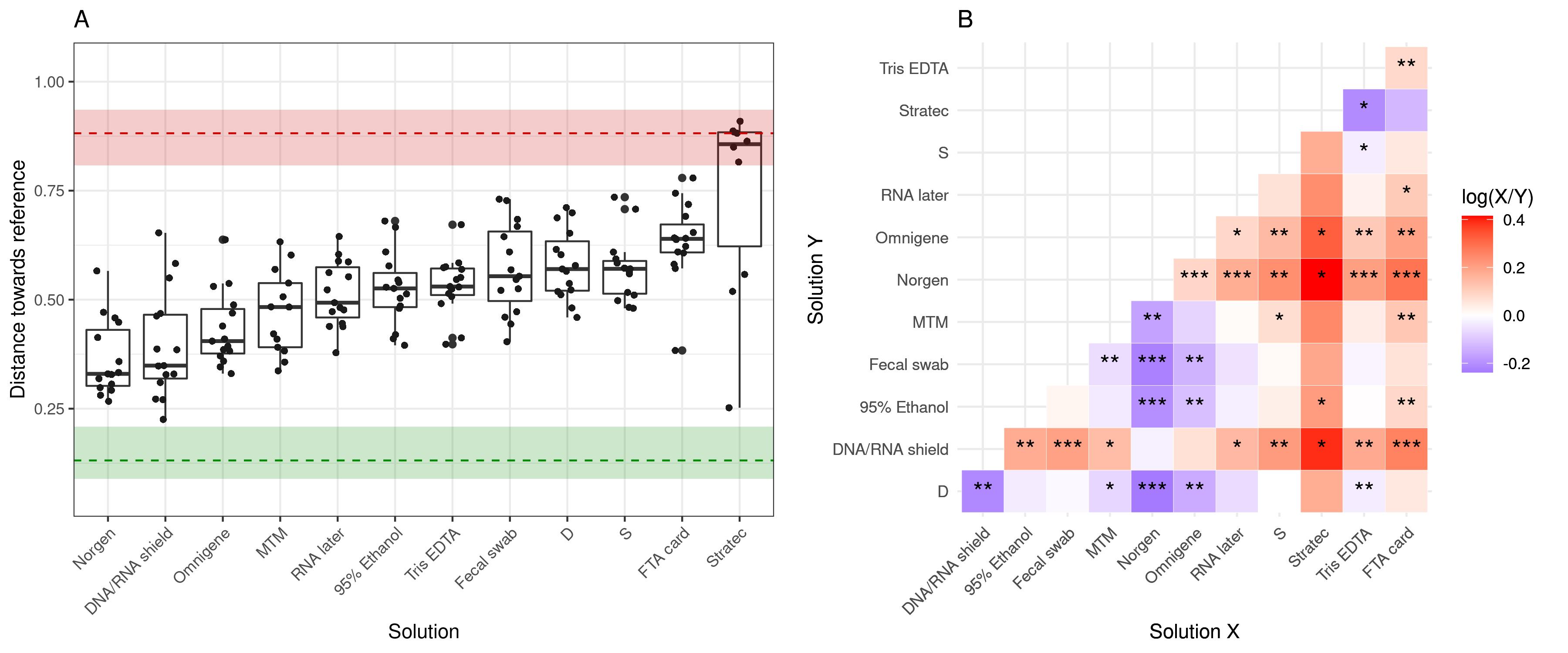

Supplement: Supplementary Figure 3 — Summary of community shifts in response to stabilizing solutions over a 14-day storage period. (A) Jaccard distance towards the reference for each participant, grouped by stabilizing solution, and the median and 5th-to-95th percentile range are shown for both interaliquot and interpatient variability. (B) A pairwise paired Wilcoxon test was performed to compare solutions with each other, the color code refers to the log10 fold change of the median performance across patients: blue means that the solution on X axis performs better, red means that the solution on Y axis performs better. Significance is shown as follows: (*) indicates fdr<0.05, (**) indicates fdr<0.01, (***) indicates fdr<0.001. [file Image_3.jpeg]

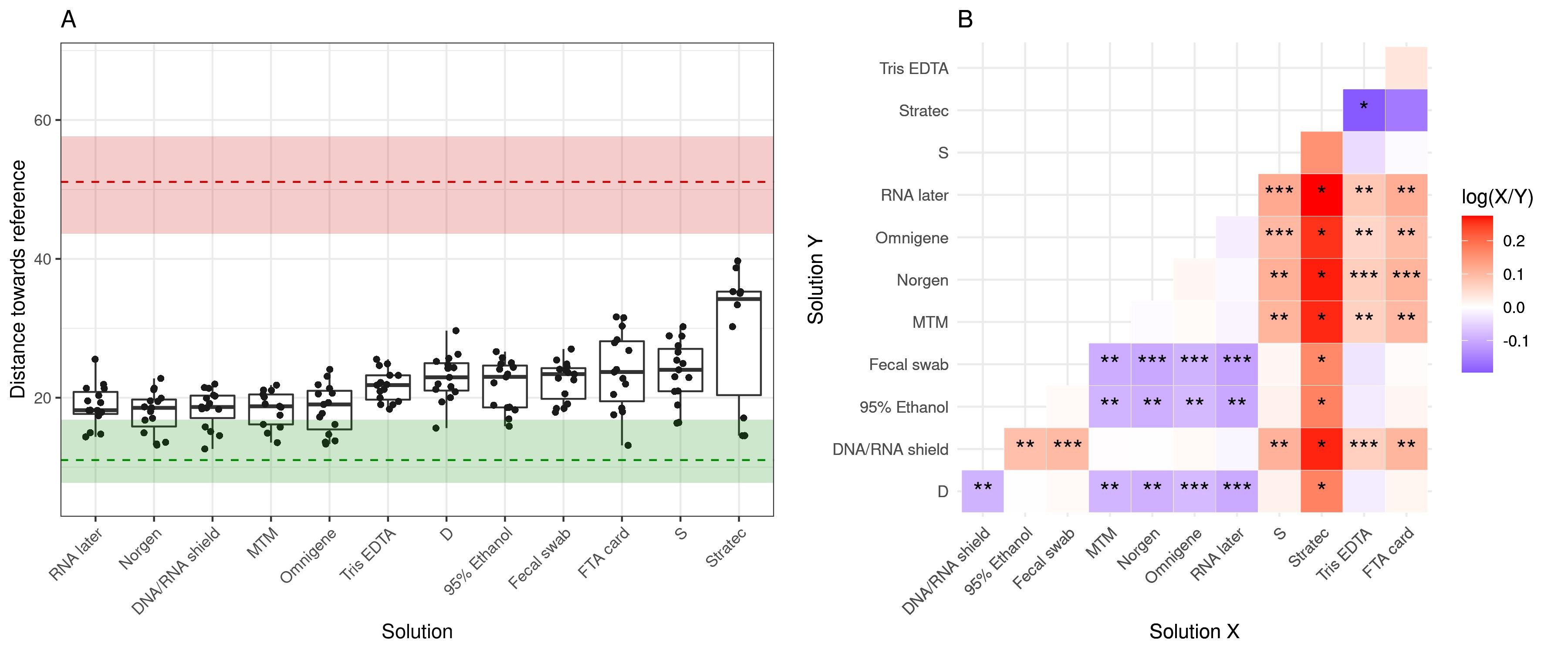

Supplement: Supplementary Figure 4 — Summary of community shifts in response to stabilizing solutions over a 14-day storage period. (A) The Aitchison distance towards the reference for each participant, grouped by stabilizing solution, median and 5th-to-95th percentile range are shown for both interaliquot and interpatient variability. (B) A pairwise paired Wilcoxon test was performed to compare solutions with each other, the color code refers to the log10 fold change of the median performance across patients: blue means that the solution on X axis performs better, red means that the solution on Y axis performs better. Significance is shown as follows: (*) indicates fdr<0.05, (**) indicates fdr<0.01, (***) indicates fdr<0.001. [file Image_4.jpeg]

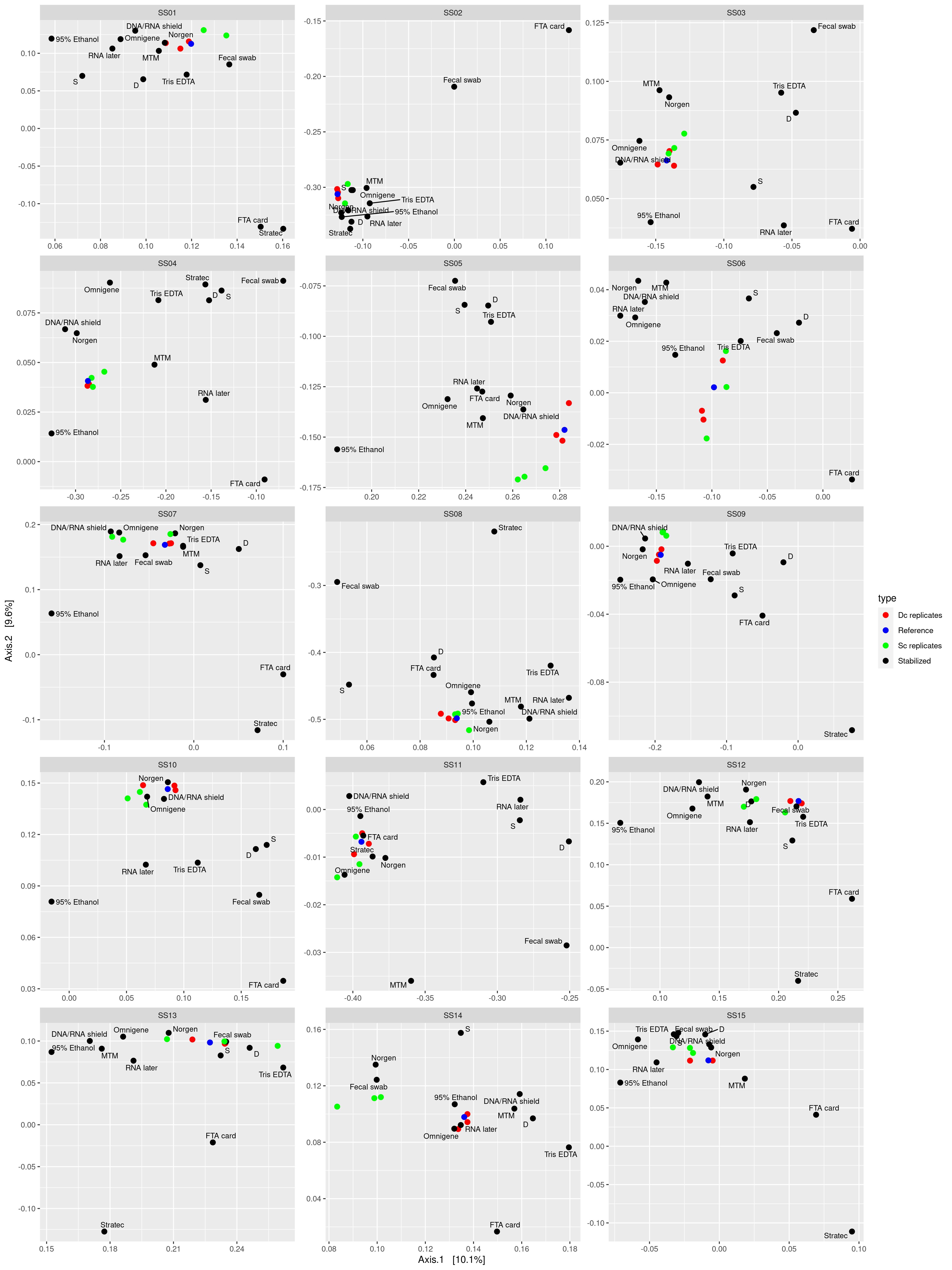

Supplement: Supplementary Figure 5 — Principal coordinate analysis (PCoA) based on Bray-Curtis distances computed from the rarefied data set. The plots are split according to samples origins, colored and labelled according to storage method. Points labelled as REF refers to the reference used for evaluation of storage methods for each patient, defined as the mean of the Dc samples. [file Image_5.jpeg]

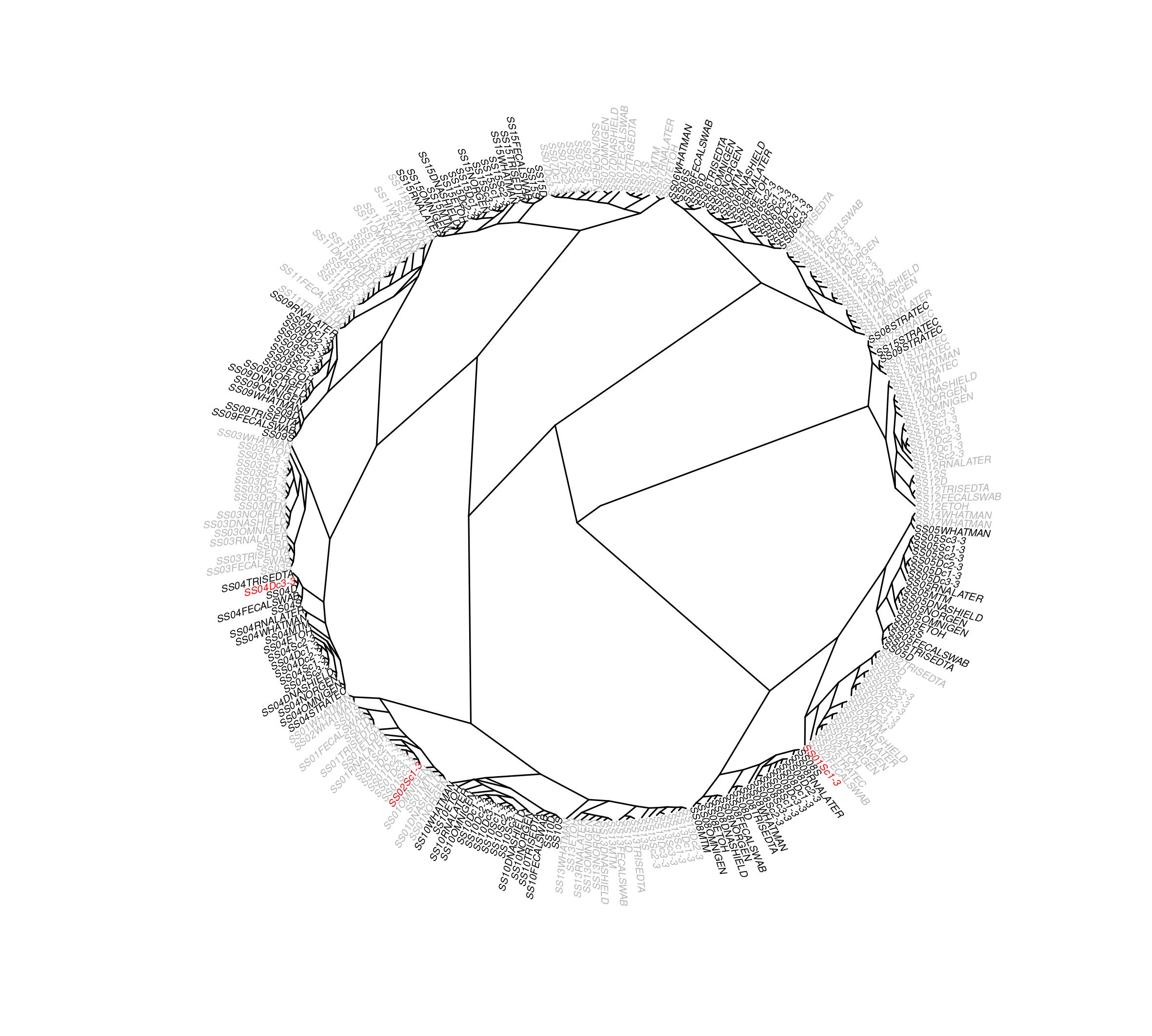

Supplement: Supplementary Figure 6 — Hierarchical clustering based on the Bray-Curtis distance matrix of all samples in the data set. The first 4 digits of the sample IDs refer to the biological origin of the fecal sample, and the remaining digits refer to the storage conditions (i.e., Sc, Dc, D, S or stabilizing solutions). Technological replicates clustered together, except for SS01Sc1-3, SS02Sc1-3 and SS04Dc3-3 (shown in red), which were excluded from downstream analysis. [file Image_6.jpeg]

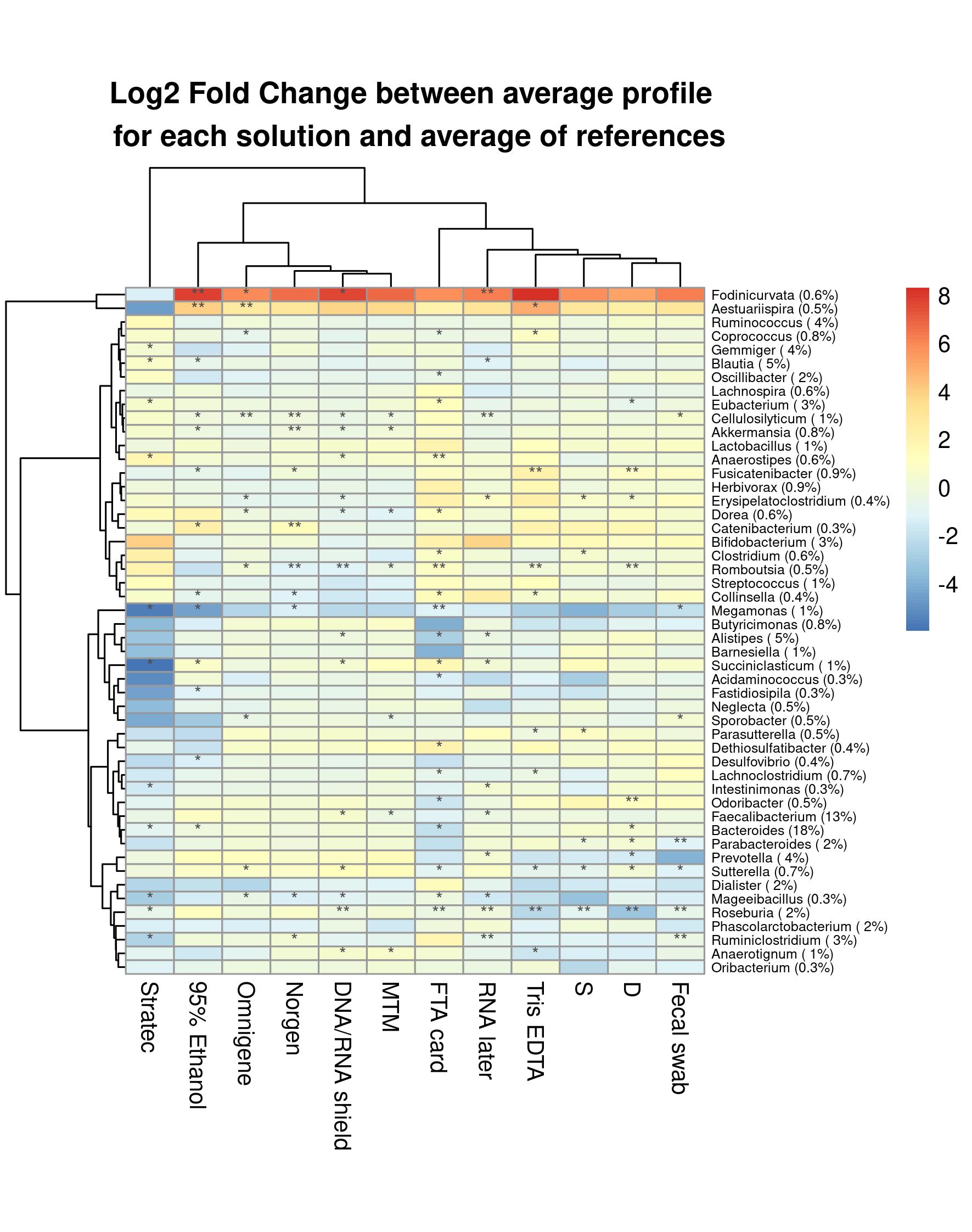

Supplement: Supplementary Figure 7 — Differentially abundant bacterial genera among samples and their references among 10 tested DNA stabilizing solutions. The median log2-fold change between average profiles and significance of the corresponding paired Wilcoxon test are shown. (*) indicates fdr<0.05, (**) indicates fdr<0.01, (***) indicates fdr<0.001. [file Image_7.jpeg]
