# Supplementary material for: Human Stool Preservation Impacts Taxonomic Profiles in 16S Metagenomics Studies
Source: Front Cell Infect Microbiol. 2022 Feb 8;12:722886. doi: 10.3389/fcimb.2022.722886 (PMC8860989; doi:10.3389/fcimb.2022.722886)
Supplement: Supplementary Table 1 — Phenotypic characteristics of the 50 most abundant genera recovered following a 14-day storage period. NEG, negative Gram strain; POS, positive Gram strain; VAR, variable Gram stain; ANAER-ST, strict anaerobic; AER-ST, strict aerobic; FAC, facultative anaerobic; MICRO-AE, microaerophile.* Proportion (%), average relative abundance of each genera within the overall dataset. [file DataSheet_1_v1.pdf]

| Genus | Proportion (%) | Gram | Oxygen status |
|---|---|---|---|
| *Bacteroides* | 18.51 | NEG | ANAER-ST |
| *Faecalibacterium* | 13.26 | NEG | ANAER-ST |
| *Alistipes* | 5.06 | NEG | ANAER-ST |
| *Gemmiger* | 4.34 | NEG | ANAER-ST |
| *Blautia* | 4.34 | POS | ANAER-ST |
| *Prevotella* | 3.86 | NEG | ANAER-ST |
| *Ruminococcus* | 3.62 | POS | ANAER-ST |
| *Eubacterium* | 3.07 | POS | ANAER-ST |
| *Ruminiclostridium* | 2.84 | POS | ANAER-ST |
| *Bifidobacterium* | 2.68 | POS | ANAER-ST |
| *Dialister* | 2.36 | NEG | ANAER-ST |
| *Parabacteroides* | 2.35 | NEG | ANAER-ST |
| *Oscillibacter* | 2.11 | NEG | ANAER-ST |
| *Phascolarctobacterium* | 1.68 | NEG | ANAER-ST |
| *Roseburia* | 1.44 | VAR | ANAER-ST |
| *Succiniclasticum* | 1.37 | NEG | ANAER-ST |
| *Streptococcus* | 1.16 | POS | FAC |
| *Cellulosilyticum* | 1.14 | VAR | ANAER-ST |
| *Lactobacillus* | 1.13 | POS | FAC |
| *Megamonas* | 1.09 | VAR | ANAER-ST |
| *Fusicatenibacter* | 1.05 | POS | ANAER-ST |
| *Barnesiella* | 1.03 | NEG | ANAER-ST |
| *Anaerotignum* | 1.00 | POS | ANAER-ST |
| *Herbivorax* | 0.93 | POS | ANAER-ST |
| *Coprococcus* | 0.83 | POS | ANAER-ST |
| *Akkermansia* | 0.77 | NEG | ANAER-ST |
| *Butyricimonas* | 0.74 | NEG | ANAER-ST |
| *Fodinicurvata* | 0.69 | NEG | FAC |
| *Lachnoclostridium* | 0.67 | POS | ANAER-ST |
| *Lachnospira* | 0.66 | POS | ANAER-ST |
| *Sutterella* | 0.66 | NEG | MICRO-AE |
| *Anaerostipes* | 0.61 | POS | ANAER-ST |
| *Aestuariispira* | 0.6 | NEG | AER-ST |
| *Dorea* | 0.57 | POS | ANAER-ST |
| *Neglecta* | 0.55 | POS | ANAER-ST |
| *Parasutterella* | 0.53 | NEG | ANAER-ST |
| *Clostridium* | 0.53 | POS | ANAER-ST |
| *Sporobacter* | 0.52 | POS | ANAER-ST |
| *Odoribacter* | 0.48 | NEG | ANAER-ST |
| *Erysipelatoclostridium* | 0.47 | POS | ANAER-ST |
| *Dethiosulfatibacter* | 0.47 | NEG | ANAER-ST |
| *Romboutsia* | 0.46 | VAR | ANAER-ST |
| *Desulfovibrio* | 0.44 | NEG | ANAER-ST |
| *Collinsella* | 0.41 | POS | ANAER-ST |
| *Acidaminococcus* | 0.39 | NEG | ANAER-ST |
| *Fastidiosipila* | 0.31 | POS | MICRO-AE |
| *Oribacterium* | 0.3 | POS | ANAER-ST |
| *Catenibacterium* | 0.29 | POS | ANAER-ST |
| *Mageeibacillus* | 0.27 | POS | ANAER-ST |
| *Intestinimonas* | 0.27 | POS | ANAER-ST |
